# Multiple diseases and polypharmacy in the elderly: challenges for the internist of the third millennium

**DOI:** 10.15256/joc.2011.1.4

**Published:** 2011-12-27

**Authors:** Alessandro Nobili, Silvio Garattini, Pier Mannuccio Mannucci

**Affiliations:** ^1^Istituto di Ricerche Farmacologiche ‘Mario Negri’, Milan, Italy; ^2^Scientific Direction, IRCCS Cà Granda Foundation Maggiore Policlinico Hospital, Milan, Italy

**Keywords:** adverse drug events, aging, geriatrics, internal medicine, multimorbidity, polypharmacy

## Abstract

The pattern of patients admitted to internal medicine wards has dramatically changed in the last 20–30 years. Elderly people are now the most rapidly growing proportion of the patient population in the majority of Western countries, and aging seldom comes alone, often being accompanied by chronic diseases, comorbidity, disability, frailty, and social isolation. Multiple diseases and multimorbidity inevitably lead to the use of multiple drugs, a condition known as polypharmacy. Over the last 20–30 years, problems related to aging, multimorbidity, and polypharmacy have become a prominent issue in global healthcare. This review discusses how internists might tackle these new challenges of the aging population. They are called to play a primary role in promoting a new, integrated, and comprehensive approach to the care of elderly people, which should incorporate age-related issues into routine clinical practice and decisions. The development of new approaches in the frame of undergraduate and postgraduate training and of clinical research is essential to improve and implement suitable strategies meant to evaluate and manage frail elderly patients with chronic diseases, comorbidity, and polypharmacy.

Journal of Comorbidity 2011;1:28–44

## Introduction

The pattern of patients admitted to internal medicine wards has dramatically changed in the last 20–30 years. The internist used to see patients mainly complaining of illnesses affecting only one organ or apparatus [1]. They had been trained in medical school and during postgraduate specialization to acquire a broad knowledge and an holistic approach to diagnosis and treatment in order to efficiently tackle the varied clinical problems presented by relatively young patients usually suffering from a single disease [1–3]. This situation changed in the last part of the 20th century, when tremendous developments in health technology made it difficult for most internists to follow progress and become proficient in the advances that marched at a fast and often overwhelming pace [2, 3]. This led to the birth or development of various subspecialties of internal medicine (such as cardiology, gastroenterology, pulmonology, and others) that had tremendous impetus and increasing popularity in the community, and hence among healthcare planners. The growth and appeal of subspecialties was paralleled by a period of uncertainty about the role and mission of general internal medicine, and in many instances, hospital medical wards had to yield space to specialized units [4, 5]. What has dramatically altered this pattern in the last few years? The fact that the internist had to deal increasingly more with the management of elderly people with multiple chronic diseases rather than with young people with single diseases.

## Population aging, chronic diseases, and multimorbidity

Elderly people are now the most rapidly growing part of the patient population worldwide, thanks to more focus on primary prevention of diseases and improvements in healthcare for the younger ill patient [6]. A century ago, one individual in 20 was aged 65 years or over, now one in six is, and by 2050 it is expected to be one in four. Individuals aged 80 years or more are the fastest growing section of the population and are expected to reach nearly 30% of the overall population in the richest nations by 2050 [7, 8].

The process of aging involves a continuum of changes in biological, functional, psychological, and social parameters that vary, depending on genetic factors, age-related vulnerability, and differences in organ function and reserves. Table 1 summarizes the main age-related changes in organ and system functions [9–11].

Aging seldom comes alone: it is often accompanied by chronic (multiple) diseases, comorbidity, disability, frailty, and social isolation [8, 10]. It is unusual for elderly patients to have only one disease affecting only one organ or apparatus [12–14]. Even though, for example, acute pneumonia may be the ultimate cause of hospital admission for an 80-year-old woman, she may very often also complain of, for instance, concomitant diabetes, heart failure, osteoporosis, anemia, and hypertension. Organ subspecialists sometimes find it difficult to tackle all these different diseases, which are unlikely to be seen concomitantly in the younger patients they are usually accustomed to caring for [15–17]. Accordingly, the holistic approach of the internist to patient healthcare has become increasingly more important, and the role and visibility of internal medicine has been magnified.

Multimorbidity in the elderly has been estimated to range from 55 to 98% [13], and is highest in the very old, in women, and individuals belonging to low socioeconomic classes [13, 18]. Although multimorbidity often simply involves the co-occurrence of two or more diseases, the distribution, combination, and development of different diseases (clustering) need to be better understood, as well as the mechanisms leading to the co-occurrence of diseases and the natural history of multimorbidity [13, 19]. In assessing these individuals, attention must be paid to genetic and biological factors, lifestyles, socioeconomic determinants, and how these factors interact to determine multimorbidity [13, 20–23].

The lack of well-designed clinical studies recruiting these patients limits the availability of evidence-based information on the effect of multiple drugs on such clinically relevant outcomes as functional and cognitive decline, quality of life, adverse events, and mortality [24–27]. Most clinical research projects in internal medicine still focus on the disease-oriented approach, which does not take account of the complexity and overlapping health and social problems of elderly patients [28, 29]. Despite these limitations, over the last few decades, many clinical care models and interventions have been developed and tested for patients with multimorbidity, especially in geriatric settings, and have been reviewed by Boult and colleagues [30].

## Polypharmacy and medication-related problems in the elderly

The prescription and use of multiple drugs to deal with concomitant multiple diseases is known as polypharmacy [31–33]. Regardless of the definition, the high prevalence of polypharmacy with aging may lead to an increased risk of inappropriate drug use, under-use of effective treatments, medication errors, poor adherence, drug–drug and drug–disease interactions and, most importantly, adverse drug reactions [34–39]. The latter are usually related to the established fact that elderly people are often frail and highly sensitive to pharmacotherapy, because of changes in pharmacokinetic and pharmacodynamic parameters [40, 41] (Tables 2 and 3) and impairment in many organ functions (Table 1) [43].

Polypharmacy is an important risk factor for inappropriate medication prescribing [35, 39, 44], which is very frequent among elderly people [35, 45]. Certain drugs are considered inappropriate or potentially inappropriate in older patients not only because of the higher risk of intolerance related to adverse pharmacokinetics or pharmacodynamics or drug–disease interactions but also because they are prescribed at too high dosages or for too long [46]. A European study involving 900 consecutive elderly patients admitted to university teaching hospitals in six countries found that potentially inappropriate prescribing ranged from 22 to 77%, depending on the criteria used [47]. However, an understated aspect of inappropriate prescribing in elderly people is also the omission of medications known to be effective in patients with an adequate life expectancy and good quality of life, because of lack of knowledge and fear of adverse drug reactions, in addition to other irrational reasons [35–37, 48–50]. The OLDY (OLd people Drugs and dYsregulations) study found that more than 40% of elderly patients were ultimately undertreated for such frequent and severe clinical ailments as heart failure, myocardial infarction, atrial fibrillation, osteoporosis, pain, and depression [51]. Moreover, polypharmacy is often an adverse consequence of the so-called ‘prescribing cascade’, which involves the clinician’s failure to recognize a new medical event as an adverse drug reaction [52, 53]. In this case, another drug is unnecessarily prescribed to treat the adverse event instead of withdrawing the drug responsible, creating a vicious circle and adding further risks.

Among hospitalized elderly patients, the prevalence of polypharmacy ranges from 20 to 60%, perhaps reflecting different criteria in the selection of patients and collection of medication data [35, 54–57]. For instance, in the REPOSI (Registro Politerapie SIMI) study, a registry based on an Italian network of 38 internal medicine wards, 52% of patients aged 65 years or older were taking five or more drugs at hospital admission. This had risen to 67% at discharge: the number of diseases, occurrence of an adverse event during hospitalization, length of hospital stay, and the presence of chronic diseases (such as hypertension, coronary artery disease, atrial fibrillation, heart failure, presence of chronic obstructive pulmonary disease, osteoporosis/osteoarthritis, and chronic renal failure) were predictors of polypharmacy at discharge [54].

Polypharmacy can also negatively influence medication adherence (compliance) [58–62]. Among elderly people, non-compliance has a prevalence of 25–75%, and the likelihood rises in proportion to the number of drugs and daily doses prescribed [58, 61, 62]. Poor adherence often becomes more marked with age, in relation to problems such as the complexity of the therapeutic regimen, visual or hearing impairment, functional and cognitive deterioration, depression, disease burden, and social isolation [58, 60–63]. Therapeutic complexity, number of different prescribers, more visits to pharmacies and lower refill consolidation have been associated with poor adherence and early discontinuation of long-term treatments. Differences in drug adherence may also be related to the days of week and the dosing regimen. For instance, failure to take a dose of a antihypertensive drug is more common at the weekend, and morning doses are more likely to be taken accurately than evening doses [64]. Non-adherence or poor adherence may result in progression of the disease, hospital admissions, and a higher healthcare cost. One study showed that 11% of hospital admissions of elderly people aged 65 years or older were the result of non-adherence and this reached 26% in those aged 75 years or more [65].

In elderly people, polypharmacy has been associated with many adverse clinical outcomes, such as drug interactions and adverse drug reactions, disability and cognitive impairment, falls and fractures, malnutrition, hospitalization and institutionalization, mortality, and rising healthcare costs [35, 37, 46, 66–76]. The increasing risk of adverse drug reactions may be related either to direct adverse effects of one or more of the prescribed drugs or to pharmacological interactions among them. A European study found that 46% of 1,601 elderly patients from six countries had at least one potentially clinically significant drug interaction [77]. The number of drugs taken is closely related to the risk of adverse drug reactions, independent of clinical diagnoses [74]. In addition, the risk of falling is positively associated with the number of drugs, irrespective of age and level of disability, particularly when elderly patients are taking benzodiazepines, diuretics, and anticholinergic agents [72].

## Limitations of guidelines in elderly people

The decision to prescribe a drug is often based on a disease-oriented approach that stems from guideline recommendations for each single symptom, disease, or clinical problem [24, 25, 28]. This paradigm of care focused on a specific disease and closely related comorbidities can be implemented easily in younger adults, but has many limitations in older patients, because it fails to take into account age-related changes in pharmacokinetics and pharmacodynamics, coexistence of other acute or chronic diseases, use of multiple drugs, risk of drug–drug or drug–disease interactions, cognitive status, and disability [46, 78, 79]. The dosages and effects of medications, beneficial or adverse, are definitely different in the elderly than in younger patients, the latter population being typically and almost exclusively enrolled in randomized clinical trials designed for drug licensing.

The evidence on which clinical guidelines are based usually stems from randomized clinical trials or meta-analyses, which are often biased by the exclusion or under-representation of elderly people, especially those affected by multimorbidity and receiving polypharmacy [24, 80–84]. A recent analysis of patient enrollment in clinical trials for cancer drugs found only 20% and 9%, respectively, of patients older than 70 and 75 years, compared with 46% and 31% for the whole cancer population in the USA [82]. Another study showed that despite the high prevalence of heart failure in older patients, more than 40% of clinical trials had one or more poorly justifiable exclusion criteria that limited the inclusion of elderly patients [84]. In most randomized clinical trials, sample size, duration, and co-prescribed drug therapies are often tailored to the target disease, and geriatric problems, such as disability, cognitive impairment, multimorbidity, life expectancy, and socioeconomic difficulties, are seldom considered [24, 25, 27, 80].

These limitations make it difficult to extrapolate the results of clinical trials and the resulting guideline recommendations to older people. For instance, if a clinician applies the relevant guidelines to a woman aged 79 years with hypertension, type 2 diabetes mellitus, chronic obstructive pulmonary disease, osteoarthritis, and osteoporosis, the patient should be taking 19 daily doses of 12 different drugs at five different times of the day, with a high risk not only of poor adherence but also of adverse reactions from drug–drug and drug–disease interactions [28]. Reliable data on patients aged 80 years or older are still not available for many diseases seen by the internists, and benchmark mortality endpoints are often of less concern for the elderly than quality-of-life issues.

Aging and frailty can also limit access to the conventional processes of care [84, 86] and, as reviewed by Weiss [87], when frail older adults interact with the healthcare system, an incomplete or distorted understanding of frailty on the part of healthcare providers can lead to an inverse relationship between an individual’s physiologic reserves and the level of demands placed on a person by the healthcare system. In conditions of low physiologic reserve, increased demands can dissipate limited resources, leading to an amplification of physiologic inefficiency. Hearing, visual and cognitive impairments can compromise medication compliance, and living alone and economic difficulties also complicate the use of vital healthcare services and diagnostic procedures, and the implementation of healthy lifestyle recommendations. Although survival is still an important outcome for many elderly people, a recent study has shown that maintaining a good quality of life and independence was indicated as the most important health outcome by nearly 80% of 357 participants [88]. So, internists must now include in their clinical practice health outcomes oriented towards a more comprehensive care of the different needs of the elderly, such as preventing the geriatric syndrome (e.g. falls, urinary incontinence, orthostatic hypotension, delirium, and depression), management of chronic pain, disability, and cognitive decline, with the aim of reducing rehospitalization and institutionalization [13, 84, 89–93].

## How can internal medicine tackle the new challenges of an aging population?

In general, the subspecialties of internal medicine still lack a systematic approach that incorporates age-related complexities into routine clinical decision-making. For the internist, the holistic and comprehensive approach for which she/he has been trained should, in principle, make it easier to tackle the challenges of multimorbidity. Nevertheless, the internist sometimes overlooks cognitive decline, functional limitations, pain, and geriatric syndromes, which in elderly patients often influence decisions and priorities on healthcare. The internal medicine community must therefore become proficient in the standards of care peculiar to the management of the elderly, and strive to achieve those skills and insights typical of geriatricians.

Internists should be trained to use multidimensional evaluation tools that broadly explore clinical, nutritional, functional, cognitive, psychological, and socioeconomic domains, providing a global assessment of the needs of the elderly [94–100]. In this multidimensional process, critical assessment of the appropriateness of pharmacological treatments and polypharmacy-related problems should become a priority, considering the patient’s global prognosis, expected benefit and time to attain benefit of drug therapy, goals of care, and life expectancy [94, 95, 101–104]. Moreover, a more critical use of the available guidelines is needed, favoring those methods designed for tailoring clinical guidelines to the comorbidity profile of individual patients as suggested by the ‘payoff time’ model [100] or by clinical care models for patients with multimorbidity [30].

Another important goal is the periodic critical review of all the medications taken [39, 79, 95, 101]. This may help to reconsider which medications are still really needed and which could or should be discontinued. The importance of setting priorities and discontinuing drug therapies has been documented in different studies and is vital when a patient is followed by many different specialists, lives alone, takes many potentially inappropriate drugs, has poor adherence, and is approaching the end of life [102–108]. For many elderly people, when clinical and functional health deteriorates, the aggressiveness of drug therapies needs to be reconsidered and clinicians must accurately select diseases that truly merit priority for treatment with the corresponding drugs. Maintaining an appropriate prescription in older patients is a dynamic process that requires periodic reassessment of the patient’s functional and cognitive status, disease priorities, socioeconomic situations, living arrangements, formal or informal support, and life expectancy, with the aim to simplify and adjust drug therapy as needed [79, 102, 103, 106, 107]. Ample evidence supports the need to critically reassess medication appropriateness and discontinuation in elderly people [106–113]. In certain patient populations, discontinuing some drugs lowers the risk of inappropriateness, reducing adverse drug reactions and cost without jeopardizing clinical success.

## How to review the appropriateness of drug prescription

During the last few decades, much effort has been directed to improving the quality of prescribing for elderly people, and several instruments and criteria have been developed by geriatricians or pharmacists [114–128]. Table 4 summarizes the most widely cited explicit and implicit criteria. Explicit criteria are usually drug- or disease-oriented and are established by expert consensus in order to draw up lists of medications that are contraindicated or should be avoided in elderly people or those with specific diseases [114–128]. Implicit criteria are mainly based on clinical judgment and are used to assess each prescribed drug with an individualized approach, in relation to a specific indication, effectiveness, dosage, adverse effects, and costs [122–124]. Each criterion has advantages and limitations reflecting its purpose, generalizability to different countries or elderly groups, updating regularity, criteria used to measure appropriateness, presence or lack of information on failure to prescribe drugs indicated for treatment or prevention of specific diseases, and inclusion or exclusion of the most frail and vulnerable people with multiple chronic diseases [126–128].

One problem is that clinicians experience difficulties in applying these instruments in daily practice, because of lack of time, poor pharmacological knowledge, fear of discontinuing or substituting drugs prescribed by others, and scepticism toward the use of too sophisticated instruments. Table 5 summarizes some of the most commonly encountered medication-related problems, their potential risks, examples of the medication, or drug classes most frequently involved, and questions that should be routinely used in order to critically assess and check the quality and appropriateness of drug prescription.

## Is a new clinical approach and paradigm of care needed by the internist?

The current paradigm of care for the elderly admitted to internal medicine wards is based on extrapolation from conventional evidence-based guidelines for each of the multiple diseases these patients often suffer. However, there is no evidence that the evidence-based therapeutic approach to a single disease is also applicable to multiple diseases and the corresponding use of multiple drugs, because there are simply no trials of polypharmacy in patients with multiple diseases (and admittedly they are difficult to plan). Not only is evidence-based knowledge on the efficacy of polypharmacy lacking but also there is the question of assuring safety. It is therefore time for a new approach by the internist for the care of elderly people, based on a combination of problem-based and patient-oriented medicine, as summarized in Table 6 and discussed below.

- Internists should improve their skills for a comprehensive evaluation of each patient, assessing not only clinical problems but also functional, cognitive, behavioral, and socioeconomic issues [95, 97, 98]. Some standardized tools developed by specialists in geriatric medicine, such as Basic [129] and Instrumental Activities of Daily Living [130], and the Mini-Mental State Examination [130] should facilitate the assessment phase. A comprehensive assessment of the patient soon after the admission has the advantage of providing clinicians with essential information to better plan the diagnostic and therapeutic approach during hospitalization, and to assess the discharge possibilities, reducing the length of hospital stay, and the risk of adverse events.
- Decisions on diagnostic tests and care should be taken according to each patient’s age, life expectancy, goals of therapies (curative or palliative), treatment target (e.g. treatment of acute illnesses, prevention of morbidity and mortality, life prolongation, maintenance of current functional or health state, and quality of life) and the expected time until benefit is achieved [104]. Treatments for symptom relief (e.g. analgesics) or acute bacterial infections (e.g. antibiotics) usually need a short time to benefit and can be prescribed to all patients. On the other hand, drugs for primary or secondary prevention of diseases, such as antihypertensive medications or statins, that require long-term dosing to obtain benefit, should only be started in patients with an adequate life expectancy. Moreover, despite considerable uncertainty about the best use of cancer screening tests in older adults, there is the need for weighing quantitative information, such as the risk of cancer death and the likelihood of benefit–risk ratio of the screening outcomes and individual patient’s values and preferences. A framework for individualized decision-making provides a helpful example of how there is a substantial variability in the likelihood of benefit for patients of similar ages with varying life expectancy [105].
- Care should be provided in accordance with best practice, and when possible should be evidence-based. However, when no such evidence is available, clinicians should identify some reliable and realistic targets for therapies, and then monitor the patient to assess target achievement or adverse drug events [24, 25, 28, 79]. Therefore, prescriptions should not be considered a single point in time of care, but a dynamic process in which the benefits and harms of drugs are continuously monitored, managed, and reassessed over time in a comprehensive longitudinal process.
- Another important goal is the critical assessment of drugs already prescribed at the time of hospital admission and of conservative prescribing at discharge. The internist should rigorously reconsider which medications are really needed and those that could be stopped. Reasons for priorities and discontinuation are well documented [103, 106–108]. To implement these processes in daily clinical practice, clinicians may choose to use some instruments (see Table 4), or keep in mind some simple suggestions: (1) critical assessment of drug therapies should be comprehensive and include a review of medical history and physical examination; (2) all medications should be reviewed according to their indication, dosages, benefit–risk profile, expected time to benefit, patient’s compliance, adverse drug reactions and risk of drug–drug or drug–disease interactions, functional and cognitive status, and effects on the quality of life; (3) potentially inappropriate drugs should be identified and their discontinuation considered; (4) the plan of discontinuation should be defined and discussed with other clinicians (the general practitioner should be informed) and communicated to the patient and/or the caregiver; (5) the patient should be followed up after discontinuation for beneficial or harmful effects.
- Discontinuation should be guided by a review of medication-related problems [38, 39, 46, 111] (see Table 5) and the pharmacological characteristics of drugs to be stopped, in order to avoid adverse events related to drug withdrawal (e.g. agitation, anxiety, confusion, delirium, or insomnia after discontinuation of a benzodiazepine), exacerbation of the condition for which the drug was originally prescribed (e.g. worsening of palpitations after withdrawing digoxin for heart failure), or the appearance of new symptoms (e.g. anxiety, insomnia, hallucinations, or depression after discontinuation of baclofen). Discontinuation may also be appropriate when lifestyle changes and behavioral interventions are able to replace pharmacologic treatment. There is evidence that non-pharmacologic interventions are preferred as initial treatment for a range of diseases too commonly treated with drugs (e.g. diabetes, hypercholesterolemia, hypertension, arthritis, insomnia, depression, and back pain). Thus, internists should become more skilled and effective at recommending smoking cessation, diet changes, exercise, physical therapy, and psychotherapy when appropriate.
- To overcome the new challenges of the aging population, the internist cannot work in isolation, because team care is essential to provide high-quality care for patients with multiple chronic diseases and polypharmacy [132, 133]. Although clinicians are poorly trained to work in teams and are often reluctant to delegate parts of care involving other professionals (clinical pharmacologists, geriatric nurses, nutritionists, physical therapists, psychologists, and social workers), a team approach should boost the efficacy and comprehensiveness of the clinical evaluation and therapeutic choices.
- Other important topics are coordination among clinicians and caregivers, and improvement in terms of communication of clinical and therapeutic decisions for the elderly [134, 135]. Thus, in the absence of electronic health records comprehensively covering the whole healthcare system and all the clinicians involved in the care of elderly people, a close relationship with the family, primary care physician and social workers is essential at hospital admission and discharge [136]. Coordination of care requires discussion, assessment of available resources, compromises and negotiations between all parties. Well-coordinated information should be provided to the family, spouse, caregiver and all the persons involved in a patient’s care, without undermining the patient’s autonomy and right to make informed choices [137].
- Communication and transparency between all providers of care and the health and social services are also essential for personalized healthcare choices [136, 138, 139]. Coordination and communication should improve the transfer of hospital care details across different hospitals, between hospital units, and at discharge when the patient goes home or to an institution. In these situations, reinforcing coordination and communication is essential to reduce patient’s stress, confusion, and agitation, and to improve such outcomes as long-term adherence to care, rates of re-hospitalization, and quality of life [138–140].
- An important topic is the incorporation of end-of-life issues in the routine care [93]. Planning end-of-life care for patients with untreatable diseases is likely to help them to accept the inevitability of death as part of the human life cycle, relieve the feeling of isolation, reorient therapeutic choices away from treatments that may no longer be useful, and focus on less-aggressive and cost-effective alternative approaches, such as homecare, home–hospital, and hospice.

## What changes are needed in the training of internists and in research?

Training of new internists and clinical research are essential components in order to improve and implement any new strategy of evaluation and management of the complexity and frailty of elderly patients with multiple diseases and polypharmacy. Learned societies of internal medicine and postgraduate schools should emphasize all the aforementioned problems related to comorbidity and include these topics in the training of specialists and in continuing medical education for specialized internists.

Research is vital to establish the best strategies of care for elderly patients admitted to internal medicine wards. Registries of older patients, designed to collect data and information with the goal of studying their comorbidity, polypharmacy, and complexity of care should help us better understand the global effects of therapies on clinical and functional outcomes. This evidence might serve as a practical basis for planning randomized controlled trials to assess how the different numbers and combination of drugs in different groups of patients, stratified according to identified disease clusters, affect mortality, disability, quality of life, and health or social care utilization. These studies should aim to compare the outcomes of various treatment regimens for those diseases that are more common in elderly populations and to assess the clinical effect and the adverse events of complex drug regimens in high prevalent clusters of diseases. A recently published article has analyzed the steps needed for enhancing the applicability of comparative effectiveness research to patients with multiple chronic diseases [25].

Research should also study the clinical burden of drug–drug interactions associated with the complex regimens for older person exposed to many drugs at the same time. These studies should examine how these multiple drugs interact globally and influence the overall benefit–risk profile of healthcare. Finally, there is the need to rethink the approach currently used to produce guidelines. In spite of the lack of detailed evidence of the complexity of elderly people with multimorbidity and polypharmacy, an effort to include and discuss these topics should be made, collecting data from registries, observational studies, or qualitative research.

## Conclusions

Modern health and social care now faces the growing challenges of rapidly aging populations as a result of the great advances made in public health, medical and pharmacological research, and preventive medicine. Internal medicine and internists are called to play a primary role in promoting a new integrated, comprehensive approach to the care of elderly people that should incorporate the complexity of age-related issues into routine clinical practice and decision-making. The internists of the third millennium must extend their paradigm of care beyond their specialty and embrace a multisystem approach, taking account of age-related changes, functional and cognitive impairment, comorbidities, polypharmacy, psychological factors, socioeconomic factors, and personal preferences. This shift is essential for individualized care of older people, for more rational and conservative drug prescribing, and to innovate evidence-based medicine with specific attention to clinical outcomes and patient satisfaction.

Most importantly, the novel approach that the internist should develop in order to optimally provide healthcare to the elderly – for the many reasons set out in this article – is also governed by the global financial crisis that is affecting the whole world. Because it appears inevitable that some degree of rationing of the ever more limited resources for healthcare will occur in the second decade of the third millennium, a more rational approach to the medical treatment of the elderly might not only help to reduce the cost of polypharmacy but could also save money in terms of less hospital admissions for adverse effects.

## Figures and Tables

**Table 1:** Main age-related changes in organ systems.

Organ system	Effects of aging	Prescribing implications
Body composition	Progressive reduction in total body water and lean body massIncrease in body fat	
Cardiac and peripheral vascular system	Heart changes (stiffening, reduced muscle strength) Reduction in the intrinsic heart rate Atherosclerosis and loss of elasticity of vessel walls	Higher systolic arterial pressure Increased impedance to left ventricular ejection Left ventricular hypertrophy and interstitial fibrosis Reduced response to postural changes Increased heart rate
Central nervous system	Increased sensitivity Decreased blood flow Decline in receptors and pathways (fewer brain cells and connections)	Enhanced response to CNS agents Slower mobility and voluntary motor activity Delirium
Gastrointestinal	Decreased secretion of hydrochloric acid and pepsin Dysfunction in GI motility Decreased GI blood flow Reduction in liver volume and blood flow	Constipation Reduced absorption and metabolism of several drugs
Immune system	Decreased immunity to diseases Greater susceptibility to infections	Increase in antibiotic use
Musculoskeletal	Loss of muscle tissue Osteoarthritis Osteoporosis	Increased use of analgesic and anti-inflammatory drugs Increased risk of falls and fractures
Renal	Reduction of renal mass and blood flow Decline in GFR	Prolonged effects of drugs poorly excreted by the kidney
Respiratory	Vital capacity and FEV may decline with age Increased rigidity of chest wall Reduced thorax muscle strength and endurance	Loss of strength and endurance of lungs with some drugs
Sensory	Visual impairment, thickening and yellowing of the lens of the eye Hearing impairment, loss of sensitivity for high-frequency tones and of discrimination of similar pitches Decline in the ability to taste and smell	Reduced adherence to drug therapies

CNS, central nervous system; FEV, forced expiratory volume; GFR, glomerular filtration rate; GI, gastrointestinal.

**Table 2:** Main age-related changes in pharmacokinetics.

	Pharmacokinetic changes^**a**^	Clinical implications
Absorption	Decrease in number of gastric and parietal cell (decrease secretions, e.g. saliva, gastric – and increase in gastric pH, achlorhydria or hypochlorhydria) Reduced gastric motility and sphincter activity (delayed gastric emptying) Decrease in mesenteric blood flow by up to 40–50%, mucosal atrophy Impaired active transport system Decrease in hepatic blood flow, less first-pass removal	Acid labile agents at normal doses may elicit a greater response (e.g. penicillins, erythromycin, levodopa) Impaired absorption of drugs requiring active transport (e.g. calcium, folic acid, vitamin B12, iron) If gastric empty is delayed, the rate but not degree of absorption may decrease Bioavailability of drugs undergoing extensive first-pass metabolism (e.g. propranolol, labetalol, morphine) may significantly increase
Drug distribution	Blood flow Plasma protein binding (decrease in serum albumin, increase in ?-1-acid glycoprotein). Acid drugs (e.g. diazepam, phenytoin, warfarin, salicylic acid) bind principally to albumin, while basic drugs (e.g. lignocaine, propranolol, quinidine, imipramine) bind to α-1-acid glycoprotein Body composition (10–20% reduction in total body water; 25–30% reduction in lean body mass; increase in body fat [males 80% and females 50%])	Water-soluble drugs (e.g. digoxin, theophylline, morphine, aminoglycosides, ethanol) tend to have smaller Vd, resulting in higher serum levels Lipid-soluble drugs (e.g. chlormethiazole, diazepam, lorazepam, oxazepam, lignocaine, thiopental) have a greater Vd, resulting in lower serum levels The reduction in Vd for water-soluble drugs tends to be balanced by a reduction in renal clearance, with little net effect on elimination half-life Changes in Vd affect the amount of drug needed for a loading dose or time needed to achieve steady-state (caution with CNS drugs such as benzodiazepines) Changes in protein binding might be clinically relevant only for drugs with a small Vd and a narrow therapeutic index. The initial transient effect of protein binding on free plasma concentration is rapidly counterbalanced by its effects on clearance
Metabolism	Liver drug clearance depends on the liver’s capacity to extract drugs from the blood passing through it, and the hepatic blood flow Rate of drug metabolism is influenced by age, smoking, nutrition, diseases, drugs, hepatic function, and serum albumin Aging is associated with a 20–30% decrease in hepatic volume, and a nearly 20–50% reduction in hepatic blood flow Phase I metabolism (hydrolysis, oxidation, reduction): mainly oxidation declines with age Phase II metabolism (conjugation) is relatively unaffected by age	The reduction of liver blood flow mainly affects the clearance of drugs with a high extraction ratio, such as chlormethiazole, propranolol, lignocaine, pethidine, glyceryl nitrate, dextropropoxyphene, morphine Significant reduction in the clearance of many drugs metabolized by phase I pathways (e.g. many SSRIs, theophylline, diazepam, quinidine, piroxicam, bupropion, nefazodone, mirtazepine) Drugs metabolized by conjugation or glucuronidation are not significantly affected Although several studies concluded that the activities of several CYP species are not specifically reduced by aging, and that there are no changes in the enzyme affinity for their substrates, the effect of age on the various CYPs is still controversial The effect of age on P-glycoprotein is still under investigation
Excretion	Kidney mass decreases by 10–20%Renal blood flow declines by 1–2% per year after the age of 40 years GFR decreases by 0.75 and 1.05 mL/min/year from age 20 to 90 years The decrease in renal blood flow exceeds the decrease in cardiac output Tubular function decreases in proportion to GFRRenal function may decline by 40–50% with age	Serum creatinine often remains stable, but creatinine clearance measurements must consider the loss of lean body mass GFR can be estimated by empirical equations (Cockcroft–Gault and MDRD). In the elderly, GFR should be estimated using the MDRD formula [42] Drugs that are excreted unchanged by the kidney (e.g. aminoglycosides, digoxin, gabapentin, lithium) may accumulate even with normal doses and should be carefully monitored A reduction in renal function may significantly affect not only renally excreted drugs, but also drugs undergoing extensive metabolism in the liver The loss of tubular function is important for drugs eliminated by tubular secretion (e.g. penicillin, cimetidine, lithium)

^a^Comprehensive information on this topic is available in recent reviews [34, 35]. CNS, central nervous system; CYP, cytochrome P450; GFR, glomerular filtration rat; MDRD, Modification of Diet in Renal Disease Study equation; SSRIs, selective serotonin reuptake inhibitors; Vd, volume of distribution.

**Table 3:** Main age-related changes in pharmacodynamics.

Pharmacodynamic changes^**a**^	Clinical implications
The impact of aging on drug sensitivity or tolerance varies with the drug and the response measured The changes observed may result from alterations in drug–receptor interactions (e.g. change in the number and/or affinity of receptors), changes in post-receptor signalling or impairment of homeostatic mechanisms Age-related changes of clinical targets may affect the pharmacological response to a drug Age-related pharmacodynamic changes in the CNS and cardiovascular system have received most attention	Increased sensitivity to benzodiazepines (e.g. sedation, confusion) with risk of falls and fractures Increased sensitivity to anticholinergic drug effects (e.g. agitation, confusion, delirium, postural hypotension) Increased sensitivity to anesthetic drugs (e.g. micovaronium, pancuronium) Reduced beta-adrenoceptor function Reduced sensitivity to the effect of verapamil on cardiac conduction Reduced sensitivity to the chronotropic effect of isoprenaline Greater inhibition of synthesis of vitamin K-dependent clotting factors by warfarin

^a^Comprehensive information on this topic is available in recent reviews [34, 35]. CNS, central nervous system.

**Table 4:** Main characteristics of commonly used instruments to assess appropriateness of drug prescribing in elderly people.

Author, year, country	Target age group(years)	Source of information	Method of validation	Number of statements	Domains (number of statements)
*Instruments based on explicit criteria*					
Beers *et al*. [114, 115] 1991 (first publication) 2003 (last update) USA	People aged ≥65	Published literature Beers [114, 116]	Three Delphi rounds (mail survey and face-to-face meeting), involving 12 internationally recognized experts in clinical geriatric pharmacology, geriatric medicine, pharmacoepidemiology, and psychopharmacology	68 criteria (48 on PIM; 20 on diseases or clinical conditions and drugs to be avoided in these clinical situations)	Drug–disease interactions (20) Drug–drug interactions (1) Drug duplication (0) Suggestions for alternative drugs (No) Indications on under-prescribing (No)
McLeod *et al*. [117] 1997Canada	People aged ≥65	Beers’ criteria [114], literature and national drug formulary	Two Delphi rounds (mail survey), involving 32 experts (9 geriatricians, 8 general practitioners, 8 pharmacists, and 7 clinical pharmacologists)	38 inappropriate high-risk prescribing	Drug–disease interactions (11) Drug–drug interactions (11) Drug duplication (0) Suggestions for alternative drugs (Yes) Indications on under-prescribing (No)
Zhan *et al*. [118] 2001 USA	People aged ≥65 (ambulatory patients)	33 criteria from Beers [116] on inappropriate drugs irrespective of dosage, frequency and duration of therapy	Two Delphi rounds (face-to-face meeting and conference call), involving 7 experts (5 geriatricians, 1 pharmacoepidemiologist, and 1 pharmacist)	33 drugs (11 drugs always contraindicated, 8 rarely appropriate, and 14 with some indication for the elderly)	Drug–disease interactions (0) Drug–drug interactions (0) Drug duplication (0) Suggestions for alternative drugs (No) Indications on under-prescribing (No)
Laroche *et al*. [119] 2007 France	People aged ≥75	Adapted to French practice from Beers and McLeod criteria, according to French Medicines Agency guidelines	Two Delphi rounds (mail survey), involving 15 experts (5 geriatricians, 5 pharmacologists, 2 pharmacists, 2 general practitioners, and 1 pharmacoepidemiologist)	34 inappropriate practices (29 drugs or drug classes to avoid and 5 drug–disease interactions)	Drug–disease interactions (5) Drug–drug interactions (2) Drug duplication (2) Suggestions for alternative drugs (Yes) Indications on under-prescribing (No)
Gallagher *et al*. [120] 2008 Ireland	People aged ≥65	Evidence-based medicine and clinical experience	Two Delphi rounds (mail survey), involving 18 experts (9 geriatricians, 3 clinical pharmacologists, 3 pharmacists, 2 primary care physicians, and 1 psychiatrist)	STOPP (65 criteria) (42 drugs to avoid in certain diseases, 4 drug combinations to avoid, 12 on duration of therapy, 2 on dosages, 3 drugs without indication, 2 on the need for additional therapy) START (22 criteria) (explicit indication for common diseases of elderly people)	Drug–disease interactions (39) Drug–drug interactions (5) Drug duplication (2) Suggestions for alternative drugs (No) Indications on under-prescribing (Yes)
Rognstad *et al*. [121] 2009 Norway	People aged ≥70in general practice	Beers’ criteria, Swedish drug recommendations, evidence from literature, and clinical experience	Three Delphi rounds (mail survey), involving 47 experts (14 clinical pharmacologists, 17 geriatricians, and 16 primary care physicians)	36 criteria on PIM (21 on single drug and dosages, and 15 on drug combinations to be avoided)	Drug–disease interactions (0) Drug–drug interactions (15) Drug duplication (1) Suggestions for alternative drugs (No) Indications on under-prescribing (No)
*Instruments based on implicit criteria*					
Hanlon *et al*. & Samsa *et al*. [122, 123] 1992 and 1994 USA	People aged ≥65 (use is not restricted to older persons)	Published literature, and clinical experience of clinical pharmacists and internist geriatricians	Sample of academic health professionals	10 criteria (10 questions to assess the appropriateness of each prescribed drug with specific instructions for use and operational definitions of each item)	Domain assessed: indication, effectiveness, dosage, appropriate directions, drug–drug interactions, drug–disease interactions, practical directions, costs, duplication, duration
Lipton *et al*. [124] 1993 USA	People aged ≥65	Potential drug therapy problems identified by researcher	Five meetings of a review panel involving 1 physician chairperson, 2 pharmacists, and 4 physicians	6 drug-therapy problem categories (each category provides definitions and examples)	Domain assessed: allergy, dosage (under- or over-dosage), schedule (frequency of administration), appropriateness (no indication, less than optimal choice), drug–drug interaction, unnecessary duplication

MAI, Medication Appropriateness Index; NORGEP, The Norwegian General Practice Criteria; PIM, potentially inappropriate medication; START, Screening Tool to Alert doctors to Right Treatment; STOPP, Screening Tool of Older Persons’ Prescriptions.

**Table 5:** Main medication-related problems and suggestions on how to review drug profiles in elderly people.

Problem	Risk	Examples	Questions for assessment
Polypharmacy	Use of multiple medications to treat acute and chronic conditions may expose the elderly to a high risk of drug–drug, drug–food, and drug–disease interactions and to adverse drug reactions The indication for a drug is often based on guidelines. However the guidelines are not always applicable in certain patients		Are all drugs prescribed indicated and effective?Which drug(s) could be discontinued?
Inappropriate prescribing	Inappropriate medication use (combinations and use of relatively contraindicated drugs) is highly prevalent among older people, particularly those admitted to hospital with acute illness This increases the risk of polypharmacy and adverse events, but paradoxically it can mask the undertreatment and suboptimal medication use (dose, formulation, duration) of indicated drugs Discontinue medications that are not working or are no longer needed, and avoid drugs that a patient has previously tried unsuccessfully or which caused adverse reactions	Long-acting benzodiazepines, non-selective beta adrenoceptor antagonists in chronic obstructive pulmonary disease Potent anticholinergic agents in dementia, lower urinary tract syndrome, constipation Verapamil, diltiazem, short acting nifedipine, NSAIDs, rosiglitazone in heart failure Antithrombotic agents to prevent stroke are often under-prescribed in elderly people ACE inhibitors in patients with diabetes and proteinuria ACE inhibitors and, if necessary, beta-blockers in heart failure	Is the patient taking inappropriate medications? Is the patient undertreated and are additional drugs indicated?
Dose and administration frequency	The dose of prescribed drugs often needs to be adjusted, particularly in elderly patients with renal or hepatic failure. Loss of renal function is very common among the elderly: in most people aged >80 years, renal function has declined by 50%. The best way to determine renal function is to measure creatinine clearance Age-related changes or alterations in pharmacokinetics and pharmacodynamics call for adjustments in dosage and frequency of drug administration	Examples of drugs that need adjustment in renal failure: ACE-inhibitors, cephalosporins, macrolides, penicillins, quinolones, sulfonamides, tetracycline, antivirals, antiepileptics, metformin and sulfonylureas, fluconazole, rosuvastatin, beta-adrenoceptor antagonists, methotrexate, diuretics, gout medications, H2 receptor antagonists, antiemetics, NSAIDs, morphine, tramadol, baclofen	Should the dose, dose frequency and/or drug formulation be adjusted?
Adverse drug reactions	ADRs are common in elderly people because of changes in pharmacokinetics and pharmacodynamics. They are implicated in 5–17% of hospital admission and 6–17% of elderly patients experience ADR while in hospital. Many ADRs could be prevented	Drowsiness, extrapyramidal syndrome with antipsychotic drugs Bleeding with NSAIDs and coumarins Bradycardia, hypotension, constipation with verapamil, diltiazem Nausea, bradycardia with digoxin Hypoglycemia with sulphonylurea antidiabetics Drowsiness and constipation after opioids Increased risk of falls after benzodiazepines, hypnotics, antipsychotics, antidepressants, anticholinergic agents, diuretics	What is the risk of ADRs and which ADRs are present?
Drug–drug interactions	The likelihood of DDI increases with age, multiple chronic diseases, organ failure, number (polypharmacy) and type of medications, drug with a narrow therapeutic window (ratio of desired effect to toxic effect) and number of physicians caring for the patient	Loss of renal function after ACE inhibitors and NSAIDs or potassium-sparing diuretics Risk of serious hemorrhage after coumarins and NSAIDs, metronidazole, miconazole, SSRIs Digoxin intoxication after digoxin and NSAIDs, diuretics, quinidine, amiodarone, verapamil, diltiazem Hyponatremia and gastrointestinal bleeding after SSRIs and diuretics or NSAIDs	What clinically important drug–drug interactions are to be expected?
		Postural hypotension and risk of falls after antihypertensives and vasodilators, antipsychotics, or tricyclic antidepressants Reduced antihypertensive effects after coadministration of antihypertensives and NSAIDs Bradycardia after beta-adrenoceptor antagonists and some SSRIs (e.g. fluoxetine, paroxetine) Increased phenytoin toxicity when coadministered with CYP enzyme inhibitors (e.g. verapamil, diltiazem, amiodarone, fluconazole, miconazole, ketoconazole, erythromycin, clarithromycin, sulphonamides, cimetidine, ciprofloxacin, grapefruit juice)	
Non-adherence (compliance)	As many as 50% of older people may not be taking their medication as intended. Inappropriate or poor adherence has been related to complexity of drug regimens, side-effects of medications, treatment of asymptomatic diseases, patient’s lack of conviction about the illness or the benefit of therapy, psychological problems (e.g. depression), cognitive impairment, inadequate discharge planning or follow-up, poor clinician–patient relationship, and cost of drugs, co-payment or both It is important to discuss the reasons for non-adherence and possible ways to improve it with the patient or caregiver Elderly people and their caregivers need to be involved in decisions about treatment and to receive full information about the benefit and risk of treatments		Does the patient adhere to his/her medication schedule?
Changes in medications after admission or discharge from hospital	Changes to medications are frequently made by patients and general practitioners after hospital discharge. These changes may be intentional, but unintentional changes are all too frequent Communication between hospital and primary care physician (and *vice versa*) must be improved to ensure shift in medication communication, to ensure treatment intended only as short-term, while the patient was in hospital, is discontinued on discharge, and to better understand medication changes		Has a full drug history been collected?
*General suggestions*			
Prescribing advice and patient or caregiver education	Patients or caregivers want more information on medicines. Providing written information about the indication(s), usage, potential risks, handling and storage of medicines is important to improve adherence. For each medication, the patient and caregiver should be informed of its purpose, how to take it, expected side-effects or drug–drug or drug–food interactions, and duration. Patients or caregivers should bring a complete medication list of prescribed and non-prescribed drugs to every visit		
Monitoring treatment	Medication prescribing should be viewed as an ongoing process that begins rather than ends with the initial decision, and requires a dynamic assessment in which the benefit and risk of drugs should be checked, managed, and reassessed over time. The goals of treatment monitoring are to ensure that the drugs are producing the intended effects, remain appropriate and to detect any medicine-related problems. Treatment monitoring is particularly important when a new treatment is started. A checklist of potential medication-related problems and a list of risk factors should help physicians establish when patients need to be referred for a more specialized medication review Monitoring may be improved by making better use of contacts with caregiver, primary care physician, and health and social care professional		
Medication review	Periodic in-depth evaluation of all the patient’s medication (prescribed and non-prescribed) should improve the quality and the appropriateness of drug prescribing. It can provide an opportunity to discontinue unnecessary or inappropriate drugs, and to add useful medications not currently prescribed Studies assessing the efficacy of drug regimens have generally been favorable, and have analysed interventions that included a pharmacist’s or clinical pharmacologist’s review, a team approach, or multidisciplinary critical drug evaluation Medication review should cover the following areas: explaining the reason and aim of the review; compilation of a list of all drugs used (including OTC, herbal, and homeopathic remedies); the patient’s (carer’s) perception and understanding of the purpose of each medication and how much, how often, and when they should be taken; potential or experienced side-effects; and review of any relevant monitoring tests (e.g. INR for anticoagulants, HbA1c for diabetic patients, and any significant blood tests)		

ACE, angiotensin converting enzyme; ADR, adverse drug reaction; CYP, cytochrome P450; DDR, drug–drug interaction; HbA1c, glycated hemoglobin; INR, international normalized ratio; NSAID, non-steroidal anti-inflammatory drug; OTC, over the counter; SSRI, selective serotonin reuptake inhibitor.

**Table 6:** Proposals for a new clinical approach and paradigm of care in internal medicine.

Proposal	Approach/Paradigm
Emphasize and practice a combination of problem-based and patient-oriented medicine	Promote a global approach to clinical evaluation of elderly patients with multiple diseases and polypharmacy Evaluate the overall effect of complexity and comorbidity not only as the sum of single diseases Set priorities for clinical, functional, and cognitive problems Identify realistic goals reflecting age-related risks, standards of care, available guidelines, and patient’s health expectations Consider comorbidity, life expectancy, quality of life, and disability during the clinical assessment and the benefit–risk evaluation for diagnostic and therapeutic choices Incorporate end-of-life issues in the balance for routine care, and plan end-of-life care for patients with untreatable diseases Incorporate patient’s preferences into care planning
Consider and screen for geriatric syndromes	Screen for functional and cognitive impairment, chronic pain, depression, urinary incontinence, risk of falls that limit patient’s quality of life and increase disability, frailty, and mortality Incorporate in clinical practice some simple standardized geriatric tools such as Barthel Index, Activities of Daily Living Index (ADL), and Instrumental Activities of Daily Living Scale (IADL) for assessing disability, Mini-Mental State Examination (MMSE) test for cognitive function, and Geriatric Depression Scale (GDS) for depression
Evaluate and manage pharmacological problems	See Tables 2, 3, and 5 Consider potentially treatable causes of disease, and seek to prevent rather than treat symptoms or advanced diseases Implement electronic prescribing tools with decision support and instant feed-back on prescribing risk for drug interactions, prescribing errors or inappropriate drug use
Promote and practice multidisciplinary and team care	Promote coordination and collaboration among all those caring for patients by discussing and sharing goals of care, monitoring and outcomes Improve communication with primary care physicians, social workers and persons involved in the patient’s care
Educate patients	Educate patients (or caregivers) to improve self (patient) care, lifestyle (diet, physical activity, smoking cessation), appropriate use of medications and health services (social support, home care, home monitoring)
